# Cost-effectiveness analysis of (accelerated) pre-operative versus (conventional) post-operative radiotherapy for patients with oral cavity cancer in Sweden

**DOI:** 10.1007/s10198-023-01578-7

**Published:** 2023-03-04

**Authors:** Maria Silfverschiöld, Kristin Carlwig, Johan Jarl, Lennart Greiff, Per Nilsson, Johan Wennerberg, Björn Zackrisson, Ellinor Östensson, Johanna Sjövall

**Affiliations:** 1 Department of ORL, Head and Neck Surgery, Skåne University Hospital, 221 85 Lund, Sweden; 2 Department of Clinical Sciences, Lund University, Lund, Sweden; 3 Department of Clinical Sciences, Malmö, Health Economics, Lund University, Lund, Sweden; 4 Department of Hematology, Oncology and Radiation Physics, Skåne University Hospital, Lund, Sweden; 5 Department of Oncology, Umeå University Hospital, Umeå, Sweden; 6 Department of Women’s and Children’s Health, Karolinska Institutet, Stockholm, Sweden; 7 Department of Medical Epidemiology and Biostatistics, Karolinska Institutet, Stockholm, Sweden

**Keywords:** Head and neck cancer, Oral cavity cancer, Cost-effectiveness, Economic evaluation, 110, 118, 119

## Abstract

**Background:**

Treatment for resectable oral cavity cancer (OCC) often includes combinations of surgery and radiotherapy (RT), but there is no conclusive information on the preferred treatment order. The aim of this study was to assess the costs and cost-effectiveness of two alternative treatment regimens for patients with OCC, reflecting pre- and post-operative RT, from a societal perspective.

**Methods:**

The study used data from the ARTSCAN 2 randomised controlled trial, which compares pre-operative accelerated RT with post-operative conventionally fractionated RT. Two-hundred-forty patients were included in the analysis of treatment outcomes. Direct costs were retrieved from the hospital’s economic systems, while indirect costs were obtained from national registries. Cost-effectiveness was assessed and a sensitivity analysis was performed. Overall survival (OS) at 5 years, was used as effect measure in the analysis.

**Results:**

Two-hundred-nine patients completed the treatments and had retrievable data on costs. Mean direct costs (inpatient and outpatient care) were € 47,377 for pre-operative RT and € 39,841 for post-operative RT (*p* = 0.001), while corresponding indirect costs were € 19,854 and € 20,531 (*p* = 0.89). The incremental cost, i.e., the mean difference in total cost between the treatment regimens, was € 6859 paralleled with a 14-percentage point lower OS-rate at 5 years for pre-operative RT (i.e., 58 vs. 72%). Thus, pre-operative RT was dominated by post-operative RT.

**Conclusions:**

From a societal perspective, post-operative RT for patients with resectable OCC is the dominant strategy compared to pre-operative RT.

**Supplementary Information:**

The online version contains supplementary material available at 10.1007/s10198-023-01578-7.

## Background

The incidence of oral cavity cancer (OCC) in Sweden is 11.3 per 100,000 individuals [1], but the malignancy is more common globally [2]. Treatment for resectable OCC often includes combinations of surgery and radiotherapy (RT), but there is no conclusive information on the preferred treatment order [3, 4]. This issue was partly addressed in the multicentre ARTSCAN 2 randomised controlled trial (RCT) [5].

In the ARTSCAN 2 RCT, patients with resectable OCC were randomised to either pre-operative accelerated RT or post-operative conventional RT. The primary outcome was locoregional control (LRC) and secondary objectives included overall survival (OS). LRC was similar for the two treatment regimens [5]. A trend towards better OS for post-operative RT (*cf*. pre-operative RT) was observed but failed to reach statistical significance. Median and minimum follow-up times for OS were nine and five years, respectively [5].

Apart from survival and morbidity of OCC, the societal cost is also an important consideration for its management [6]. Such costs may be described as direct (i.e., health care resources spent for work-up and treatment) or indirect (i.e., production lost due to sick-leave and early retirement). Furthermore, a cost-effectiveness analysis (CEA) may be performed to ascertain that societal resources are used efficiently [7]. Costs and cost-effectiveness per order of surgery and radiotherapy for OCC have not previously been described.

Information on outcome, morbidity, and societal costs is central for decision-makers when implementing new healthcare routines in clinical practise. The aim of this study was to assess costs and cost-effectiveness for patients with OCC and compare pre-operative with post-operative RT according to the ARTSCAN 2 RCT.

## Methods

### Patients and design

**Fig. 1:**
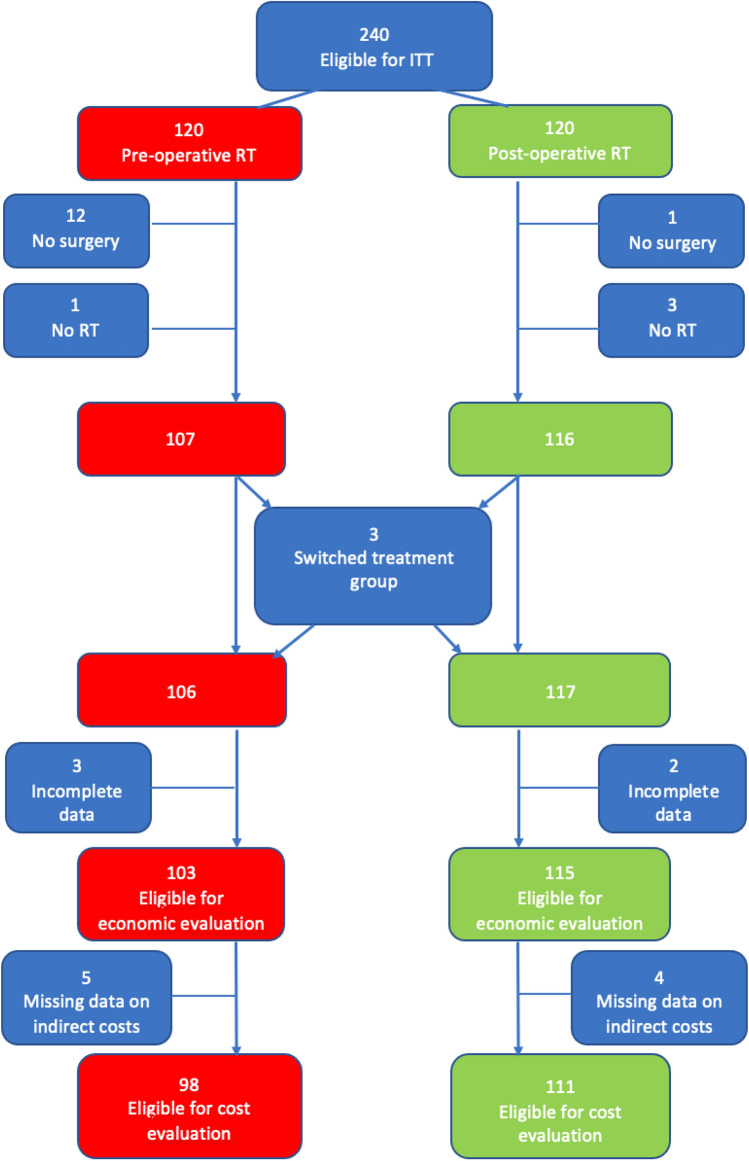
Flowchart for the 240 patients eligible for intention-to-treat population analysis. Twenty-one were excluded from the pre-operative RT group and 10 from the post-operative RT group, leaving 209 patients for the present analysis. Abbreviations: *ITT* intention to treat; *RT* radiotherapy

Two-hundred-fifty patients were randomised in the ARTSCAN 2 RCT, which involved combined therapy with surgery and RT for advanced, resectable OCC performed at six centres in Sweden from 2008 through 2016 [5]. The patients were offered to participate after the diagnostic work-up was finalised. After informed consent, they were randomised 1:1 to either accelerated RT (68 Gy in 4.5 weeks) followed by surgery or surgery followed by conventionally fractionated RT (60 Gy in 6 weeks to low-risk patients; 66 Gy in 6.5 weeks plus concomitant cisplatin to high-risk patients). The randomisation was centralised using a minimisation algorithm balancing the factors trial centre, tumour subsite (tongue or floor of mouth *vs.* gingiva or other oral subsites), and clinical stage (I–II vs. III–IV). Treatment allocation could neither be blinded to patients nor clinicians. Two-hundred-forty patients were eligible for intention-to-treat analysis (120 patients in each treatment group) [5]. Details on data availability for the present analysis are indicated in Fig. 1. A CEA was performed taking costs, and OS-rate at five years, into account.

### Cost assessment

Direct costs, specified for inpatient and outpatient care, were retrieved from the hospital’s economic systems for patients treated at Skåne University Hospital, i.e., a public, non-profit hospital and one of the six participating centres, which randomised more than a third of the patients in the ARTSCAN 2 RCT. Data on direct costs from the randomisation date and three years onwards (or until death) were assessed. Data were cross-checked against the patients’ medical records and cleared from costs produced by comorbidities. Costs for OCC were imputed for the remainder of the cohort (the other five centres), for which no such data were available, using multiple imputation adjusted for age, sex, site, clinical stage, and treatment, separately for inpatient and outpatient costs.

Indirect costs for sick-leave and early retirement due to post-treatment morbidity from the randomisation date and 3 years onwards (or until death) were retrieved from the Swedish Social Insurance Agency for the entire study population. The number of days (sick-leave and early retirement) per patient was then multiplied by an average daily salary (based on an average monthly income for all working individuals, both females and males, of SEK 35 300) plus social fees at 2019 year level (37.06%) [8]. Sick-leaves due to other illnesses were disregarded. Information on any sick-leave shorter than 14 days, which in Sweden is the responsibility of the employer and not reported to the social insurance agency, was not available.

All costs were adjusted to the 2019 year price level using the consumer price index (CPI) [9]. In addition, costs were expressed in Euro after adjustment for purchasing power parity (PPP) for 2019 [10].

### Effect measure

The primary effect measure for the ARTSCAN 2 RCT was LRC, and secondary measures, including OS, were calculated from the date of randomisation. OS at 5 years was used as effect measure in the analysis and illustrated with the Kaplan–Meier method, and differences between the two treatment groups were compared with the log-rank test [5]. Difference in OS at a fixed point in time, i.e., at 5 years as used for the CEA, was compared with the method described by Klein et al. [11].

### Cost-effectiveness analysis

The incremental cost and effect were calculated as the differences in cost and OS-rate at 5 years between pre-operative RT, i.e., the intervention, and post-operative RT, i.e., the base-case regimen. The cost-effectiveness was then estimated as the incremental cost-effectiveness ratio (ICER), which represents the cost per additional percentage point of OS.

$${\text{ICER}} = \frac{{{\text{Costs for postoperative RT}}( \text{EUR}) - {\text{Costs for preoperative RT}}(\text{EUR})}}{{{\text{OS of}}{\mkern 1mu} {\text{postoperative RT}}\left( \% \right) - {\text{OS of preperative RT}}\left( \% \right)}}.$$

Uncertainty regarding incremental costs and effects was assessed using non-parametric bootstrapping with 5000 replications [7]. For this purpose, a multivariable logistic regression was performed following Saha et al. [12], with the dependent variable “alive at 5 years” or “dead within 5 years” and the independent variables age, sex, site, clinical stage, and treatment group. The bootstrapped cost-effectiveness pairs were presented in a four-quadrant cost-effectiveness (CE)-plane with the differences between the intervention and the base-case regimen in effect on the x-axis and the difference in total cost on the y-axis [13]. The CEA largely followed the Consolidated Health Economic Evaluation Reporting Standards 2022 (CHEERS 2022) [14].

### Sensitivity analyses and subgroup analyses

A deterministic one-way sensitivity analysis was performed to evaluate the impact of the included parameters on the ICER. Costs for inpatient and outpatient care, indirect costs, and OS-rate at five years were independently varied by ± 1 standard deviation while keeping all other variables constant. In addition, analyses were performed for subgroups, i.e., based on retirement age, gender, clinical stage, and site. Finally, cost-effectiveness  was separately assessed for the patients treated at Skåne University Hospital to validate the imputation of direct costs from this subset onto the remainder of the study population.

### Statistics

Differences in cost between the regimens were analysed with independent group *t* tests using Stata/BE 17 (StataCorp LP, College Station, TX).

## Results

Out of the 240 patients eligible for intention-to-treat analysis in the ARTSCAN 2 RCT [5], 31 individuals were excluded. The reasons for withdrawal are indicated in Fig. 1. Two patients were offered to switch from pre- to post-operative RT and one from post- to pre-operative RT due to temporarily extended waiting times to start of intended treatment. Accordingly, 209 patients were available for the present analysis, i.e., 98 in the pre-operative RT group and 111 in the post-operative RT group.

Data on demographics for the study population are presented in Table 1. The characteristics were well-balanced between the two treatment regimens (Table 1).

**Table 1:** Characteristics of patients available for analysis

	Pre-operative RT *n* = 98	Post-operative RT *n* = 111	Total *n* = 209
Mean age (range)	65 (31–84)	64 (23–84)	65 (23–84)
Female/male No. (%)	32 (33)/66 (67)	43 (39)/68 (61)	75 (36)/134 (64)
Primary site No. (%)			
Tongue/floor of mouth	66 (67)	79 (71)	145 (69)
Gingiva and other oral subsites	32 (33)	32 (29)	64 (31)
Clinical stage No. (%)			
I + II	49 (50)	56 (50)	105 (50)
III + IVA	49 (50)	55 (50)	104 (50)
Smoker No. (%)			
No (never)	35 (36)	49 (44)	84 (40)
Yes (active or previous)	62 (63)	62 (56)	124 (59)
Unknown	1 (1)	0 (0)	1 (< 1)

*RT* radiotherapy

**Fig. 2:**
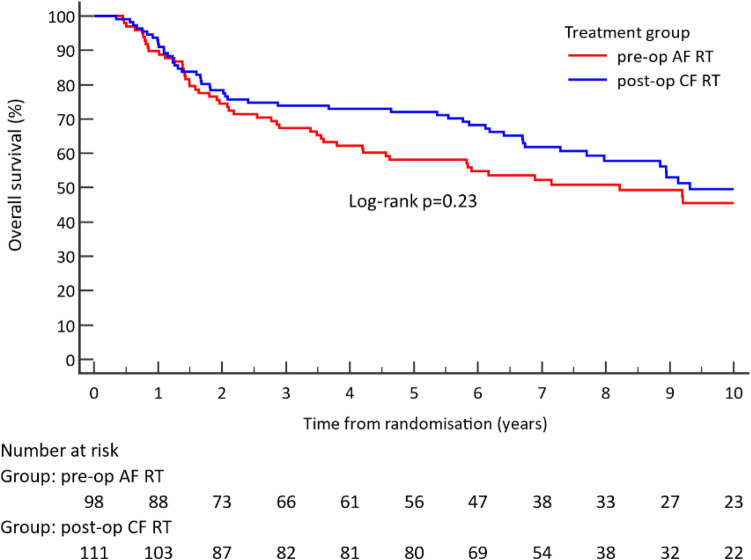
Overall survival (%) for accelerated RT (68 Gy) followed by surgery, or surgery followed by conventionally fractionated RT (60 Gy to low-risk patients; 66 Gy plus weekly chemotherapy to high-risk patients) for the population (*n* = 209). Abbreviations: *AF* accelerated fractionation; *CF* conventional fractionation

There was no difference in LRC between the treatment regimens in the present patient cohort: LRC at 5 years was 77% (95% CI 68–86) for pre-operative RT and 76% (95% CI 64–81) for post-operative RT (detailed data not shown). Likewise, a trend towards better OS for post-operative RT (*cf.* pre-operative RT) was observed: log-rank *p* = 0.23 (Fig. 2). OS, when compared at the fixed time point of five years, was however found to be significantly higher for post-operative RT (72% [95% CI 64–81]) compared to pre-operative RT (58% [95% CI 49–69)]) (*p* = 0.036). The mean life years (LY) lost during the first 5 years was 1.3 for pre-operative RT and 1.0 for post-operative RT, resulting in a mean 0.3 LY gain in favour of post-operative RT.

Direct and indirect costs are presented in Table 2. The total direct cost for work-up and treatment was less for post-operative RT (€ 39,841) compared to pre-operative RT (€ 47,377), and this difference reached statistical significance. Similarly, the subsets of direct costs representing outpatient and inpatient care were less for post-operative RT *cf.* pre-operative RT, with both subsets reaching statistical significance. The indirect costs for sick-leave and early retirement were similar between the groups.

**Table 2:** Mean (SD) direct and indirect costs per patient and treatment regimen for resectable OCC expressed in PPP-adjusted Euro (€) for 2019

	Pre-operative RT *n* = 98	Post-operative RT *n* = 111	Cost difference (95% CI)	*p* value
Direct costs	47,377 (15,753)	39,841 (15,387)	7536 (3284 to 11,788)	0.001
Outpatient care	15,935 (4270)	13,728 (4414)	2207 (1019 to 3395)	0.000
Inpatient care	31,441 (13,629)	26,113 (13,114)	5329 (1678 to 8979)	0.004
Indirect costs	19,854 (34,242)	20,531 (38,138)	− 678 (− 10,615 to 9260)	0.893
Total costs	67,231 (41,155)	60,372 (42,559)	6859 (− 4593 to 18,310)	0.239

Direct costs from the population of the Southern Health Care Region of Sweden imputed onto the remainder of the study population

*PPP* purchasing power parity; *RT* radiotherapy; *SD* standard deviation; *CI* confidence interval

**Fig. 3:**
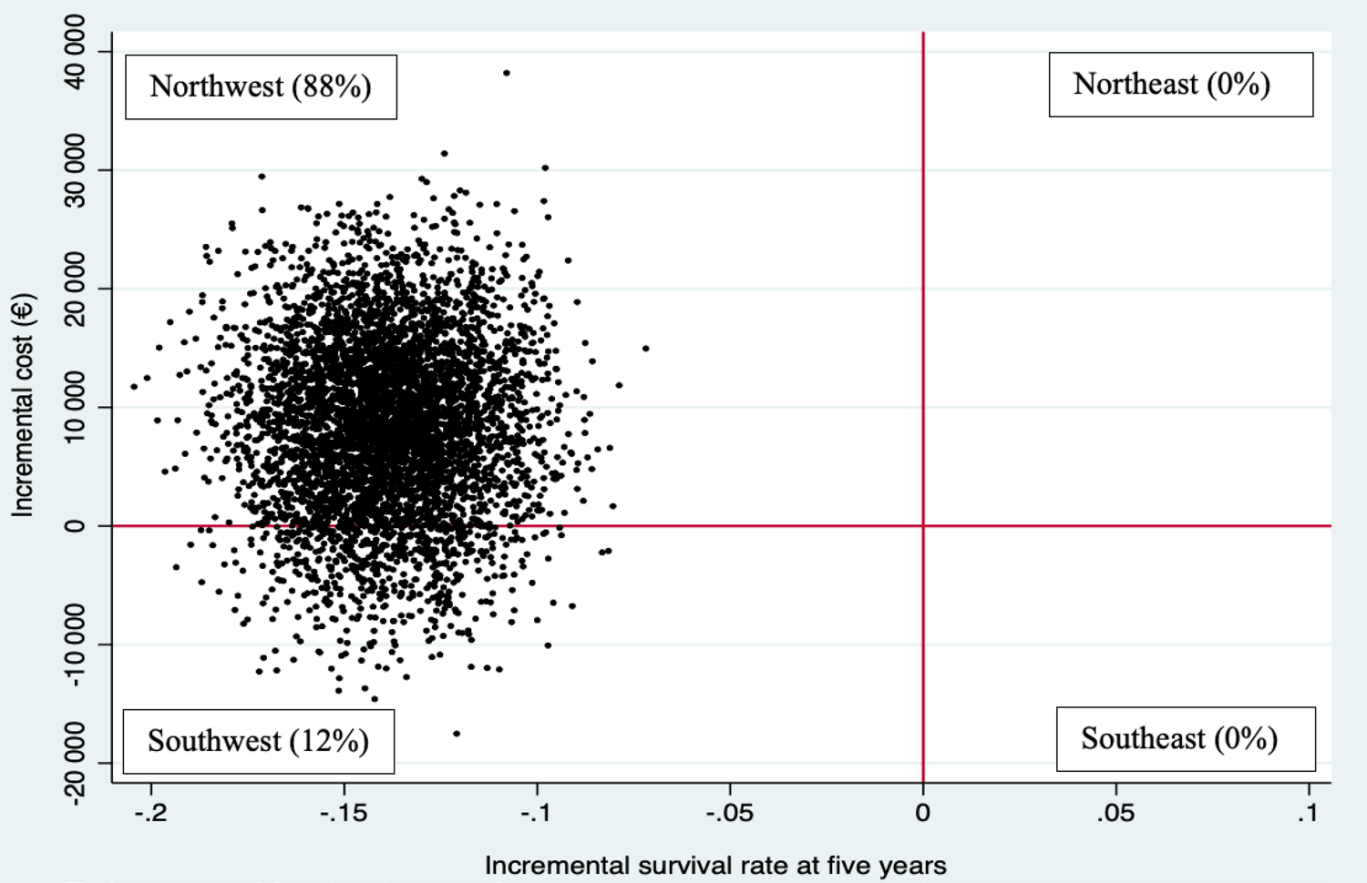
Cost-effectiveness plane (CE-plane). The different quadrants of the CE-plane show different combinations of incremental costs and effects. 88% of the ICER pairs were in the northwest quadrant, which indicates that pre-operative radiotherapy was dominated by post-operative  radiotherapy

The intervention, i.e., pre-operative RT, led to significantly higher costs and a lower probability of survival at five years (− 14-percentage points). On the CE-plane, 88% of the pairs were in the northwest quadrant, indicating that pre-operative RT was more costly and less effective, while 12% were in the southwest quadrant (less costly and less effective) (Fig. 3). Thus, pre-operative RT was dominated by post-operative RT. In the sensitivity analysis, the cost-effectiveness results were robust to variations in costs and five-year survival rate. All subgroup analyses were in keeping with the base-case analysis (Table 3).

**Table 3:** Sensitivity and subgroup analyses of the cost-effectiveness of pre-operative compared to post-operative RT

Scenario	Cost difference	Effect difference	ICER
Base case	6859	− 13.9	Dominated
Sensitivity analysis			
Outpatient costs adjusted + 1SD	6714	− 13.9	Dominated
Outpatient costs adjusted -1SD	7002	− 13.9	Dominated
Inpatient costs adjusted + 1SD	6897	− 13.9	Dominated
Inpatient costs adjusted -1SD	6819	− 13.9	Dominated
Indirect costs adjusted + 1SD	2962	− 13.9	Dominated
Indirect costs adjusted –1SD	10,754	− 13.9	Dominated
Effect-adjusted + 1SD	6859	− 9.8	Dominated
Effect-adjusted –1SD	6859	− 18.0	Dominated
Subgroup analyses			
Age			
23–65 (47/51)	2842	− 12.9	Dominated
66–84 (51/60)	8532	− 14.9	Dominated
Gender			
Female (32/43)	3232	− 10.1	Dominated
Male (66/68)	9509	− 14.5	Dominated
Clinical stage			
I/II (49/56)	10,746	− 10.6	Dominated
III/IVA (49/55)	2884	− 17.2	Dominated
Subsite			
Tongue/floor of mouth (66/79)	8348	− 14.2	Dominated
Gingiva and other oral subsites (32/32)	5889	− 13.9	Dominated

All costs are expressed in PPP-adjusted Euro (€) for 2019 and effects in percentage points. Population size for pre- versus post-op RT in paratheses

*PPP* purchasing power parity, *RT* radiotherapy, *SD* standard deviation

## Discussion

An overall analysis of the ARTSCAN 2 RCT indicates that pre- and post-operative RT are equally effective alternatives for resectable OCC regarding LRC and OS when combinations of surgery and RT are considered [5]. In addition, the present analysis based on this trial now demonstrates that pre-operative RT is more expensive and less cost-effective compared to post-operative RT.

In this study, the direct cost for pre-operative RT was significantly greater compared to post-operative RT, and it was driven by costs for inpatient as well as outpatient care. The “bottom-up” analysis of direct costs was of importance as it enabled us to clear the data from costs produced by conditions other than, or considered not associated with, OCC and its treatment. As this detailed level of information was available only for the population treated at Skåne University Hospital, these data were imputed for the remainder of the study population. This procedure was validated by comparing the cost-effectiveness  outcome between the patients treated at Skåne University Hospital and the entire study population (data not shown): our conclusions were not affected. A more detailed specification of direct costs can unfortunately not be provided, as the recordings were not uniform over the study period. However, we may speculate that the greater pre-operative RT-group costs for in- and outpatient care might reflect a more marked acute toxicity/morbidity produced by accelerated RT *cf*. conventionally fractionated RT (i.e., the post-operative treatment), which has been described in the context of head and neck cancer [15].

The present study assessed cost-effectiveness, and one-way sensitivity- and subgroup analyses were performed. In the CEA, uncertainty regarding incremental costs and effects was assessed using non-parametric bootstrapping, which is a method commonly used to describe the distribution of possible mean values [7]: it uses resampling from the data to generate new estimates of the sampling distribution. The outcome indicated that post-operative RT was the preferred/cost-effective treatment: the costs were lower and there was a difference in OS at five years between the regimens in favour of post-operative RT. However, we acknowledge that specific data on quality of life are warranted for the CEA to be fully comprehensive. Cost-effectiveness for specific patient subgroups in terms of age, gender, site, and clinical stage was also assessed, indicating that post-operative RT was even more cost-effective (*cf*. pre-operative RT) for persons over 65 years of age, men, and tongue/floor of mouth subsite. However, the subgroup results should be interpreted with caution due to small sample sizes and that stratification was not performed for age and gender.

Several studies have estimated societal costs for head and neck cancer, but few exclusively on OCC and, to the best of our knowledge, none with a focus on costs and cost-effectiveness of pre-operative RT versus post-operative RT [16, 17]. Furthermore, in general, previous cost studies on head and neck cancer have key drawbacks. First, they report only direct or indirect costs [18–24]. Second, they are “top-down” analyses of registry data, not curated against medical records [23, 24]. In contrast, the present study assesses costs specifically for OCC, including direct medical costs of work-up and treatment from a “bottom-up” perspective and indirect costs (e.g., costs due to sick-leave and early retirement), both cleared from costs of co-existing conditions. The advantage of having patient-specific data on societal costs and outcomes is that these can be further used in CEAs, such as in this study, to determine whether a treatment is justified in terms of health gains for patients with OCC.

Two previous studies focus on the impact of the order of surgery and RT in head and neck cancer, both arguing for post-operative RT [25, 26]. However, ARTSCAN 2 is the first RCT that can be analysed in this context, and the interpretation is that the regimens are equal in terms of LRC and OS [5]. In that context, the present observations on costs and cost-effectiveness may be of particular interest to surgeons/oncologists and other decision-makers when choosing the treatment order for resectable OCC: we forward a treatment order of surgery followed by RT. However, a complete analysis of complications, morbidities, and quality of life associated with OCC and its treatment is warranted and will be analysed later by the ARTSCAN 2 Study Group. Since the direct cost data obtained in this study are from a public, non-profit health care system, the cost level is likely generalisable to similar systems, e.g., the Nordic Countries.

In contrast, extrapolation to private health care and government-run insurance programmes should be avoided. The results of this study will reinforce the routine at hospitals where post-operative RT is currently standard. If the opposite is the situation, the current results, in connection with other publications of the ARTSCAN 2 study, suggest that a change of treatment order may be considered.

### Strengths and limitations

The strengths of the present economic evaluation are the preciseness of the cost data and that it is based on an RCT. A limitation is a lack of data on complications, morbidities, and quality of life. For example, such data would have enabled calculations on quality-adjusted life years. Furthermore, direct costs were available only for the patients treated at Skåne University Hospital but were imputed for the remainder of the cohort and validated by comparing the outcome between the subset and the entire study population. Moreover, sick-leave shorter than 14 days was not monitored, but we do not consider this to be a major issue since sick-leaves due to OCC generally exceed 14 days. Finally, the lack of costs for primary, palliative, and informal care, prescription drugs, and non-market productivity losses are other limitations of the study.

## Conclusions

Based on data from the ARTSCAN 2 RCT, we conclude that post-operative RT for resectable OCC is the dominant alternative compared to pre-operative RT.

### Supplementary Information

Below is the link to the electronic supplementary material.

Supplementary file1 (PDF 74 KB)

## Data Availability

The datasets generated and/or analysed during the current study are not publicly available due to privacy/integrity reasons, but are available from the corresponding author on reasonable request.

## Abbreviations

AF: Accelerated fractionation
CE: Cost-effectiveness
CEA: Cost-effectiveness analysis
CF: Conventionally fractionation
CI: Confidence interval
CPI: Consumer price index
Gy: Grey
ICER: Incremental cost-effectiveness ratio
ITT: Intention to treat
LRC: Locoregional control
LY: Life years
OCC: Oral cavity cancer
OS: Overall survival
PPP: Purchasing power parity
RCT: Randomised controlled trial
RT: Radiotherapy
SD: Standard deviation
